# Oblivion: autopsy findings of a 31-year-old man with sudden cardiac arrest, a case report of a sequalae of Kawasaki disease

**DOI:** 10.4322/acr.2021.404

**Published:** 2022-10-19

**Authors:** Daniel Fernando Gallego, Maria Eugenia Zuluaga Ruiz, Desiree Ann Marshall

**Affiliations:** 1 University of New Mexico, Office of the Medical Investigator, Albuquerque, New Mexico, USA; 2 Universidad del Valle, Grupo de Investigación en Rehabilitación de la Universidad del Valle, Cali, Valle del Cauca, Colombia; 3 University of Washington, Department of Laboratory Medicine and Pathology, Seattle, Washington, United States

**Keywords:** Coronary Aneurysm, Death, Sudden, Mucocutaneous Lymph Node Syndrome, Forensic Pathology, Coronary Disease

## Abstract

A 31-year-old man presented to the hospital after suffering a sudden cardiac arrest. Despite optimal therapy, the patient passed away. His medical history included febrile rash at age 2. At autopsy, there was aneurysmal dilation and severe coronary artery stenosis by atherosclerotic plaques and myocardial fibrosis. These findings were presumed to be due to complications of Kawasaki disease, given the remote history of severe febrile rash as a toddler and the presence of chronic coronary artery injury, recanalization, and thrombosis with ischemic heart disease leading to sudden cardiac collapse and death.

## INTRODUCTION

Kawasaki disease (KD) is a medium vessel vasculitis that particularly involves the coronary arteries and commonly affects children. The incidence rate of KD is 25-50 cases per 100,000 persons per year in the United States^1^ and is recognized as the most common acquired childhood heart disease in developed nations.^2^ The exact etiology of KD remains unknown, some hypotheses include infection due to an intracellular pathogen, such as a virus,^3^ but also a genetic component is suspected due to its higher rate in children of Asian ethnicity and in Japanese American children who have a Western diet and lifestyle.^4^ Despite appropriate treatment, in infants <6 months of age, the risk of a coronary artery aneurysm is 50% and 5% for older children.^1^ Less described in medical literature are the long-term sequelae of KD, with the persistence of the aneurysm, chronic inflammation, and pro-coagulation states with associated thrombosis and potential delayed mortality. Also, an association with hypercholesterolemia and atherosclerosis has been suggested. We present the clinical case and autopsy findings of an adult male who died of remote KD’s sequelae.

## CASE REPORT

A 31-year-old white man presented with cardiac arrest while riding his bicycle. He received bystander cardiopulmonary resuscitation (CPR) with return of spontaneous circulation (ROSC), and was transferred to a tertiary hospital (located in the Northwestern United States). On arrival, he was unresponsive, but hemodynamically stable, the electrocardiogram (ECG) was unremarkable, and the initial troponin I that was at 0.10 ng/mL (normal <0.04 ng/mL). Urgent cardiac catheterization was attempted demonstrating coronary artery aneurysms and severe stenosis. Echocardiogram revealed an ejection fraction of 45% with akinetic inferior and inferior/lateral walls.

Past medical history included hypercholesterolemia and at age 2, a severe febrile rash with associated lymphadenopathy that per the family, was diagnosed as Kawasaki disease. The febrile rash was treated symptomatically with supportive therapy (unclear if he received aspirin), and he was discharged without apparent sequelae. No medical records were available from this event, and the given information was from memory recollection of the family members.

Cholesterol profile from the admission included total cholesterol 245 mg/dL (normal, <240 mg/dL), direct low-density lipoprotein (LDL) 169 mg/dL (normal, <160 mg/dL), high-density lipoprotein (HDL) 38 mg/dL (>40 mg/dL), and triglycerides 124 mg/dL (<200 mg/dL). Per discussion with the family, the decedent did not take any medications.

Despite optimal therapy, the decedent remained comatose, suffered anoxic brain injury, and developed aspiration pneumonia with severe acidosis. He was transitioned to comfort care measures only, and died four weeks after admission.

## AUTOPSY PRESENTATION

**Figure 1:**
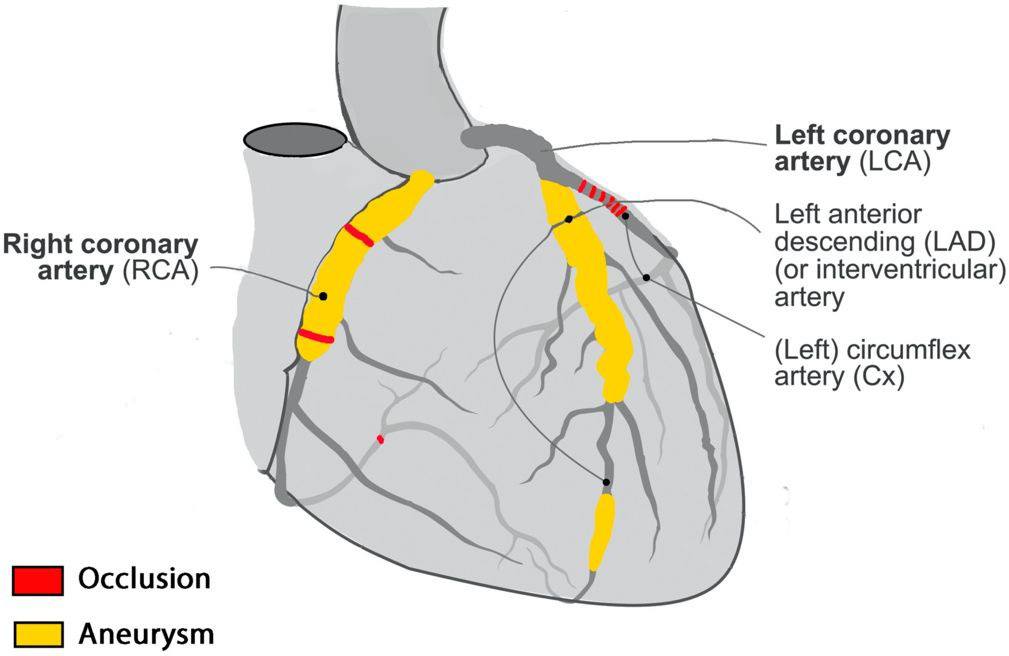
Diagram of the heart showing aneurysmal dilation of the coronary arteries highlighted in yellow and severe coronary artery atherosclerosis highlighted in red. Modified with permission from Creative Commons via Wikimedia Commons.

On autopsy, the patient was a well-developed male (height: 178 cm, weight: 69 Kg, body mass index: 21.8 Kg/m^2^). His heart weight was 375 g (RR: 233-383 gr),^5^ containing transmural scar of the left lateral/posterior wall (5 cm), transmural scars on the left posterior wall (2.5 cm) with associated myocardial thinning, and subendocardial fibrosis of the left anterior wall (2.5 cm). Coronary evaluation demonstrated multiple aneurysms admixed with varying degrees of atherosclerotic stenosis (detailed summary in Figure 1 and Table 1).

**Table 1:** Summary of gross coronary artery findings.

Coronary arteries	Aneurysmal dilation	Atherosclerosis
Left main	Not significant	Not significant
Left anterior descending	1) 3 cm in length, starting 2.5 cm from ostium. Up to cm in 1.8 cm in diameter.	Not significant
	2) 0.8 cm in length, distal portion. Up to 0.8 cm in diameter.	
Left circumflex	Not significant	1) Complete occlusion, 1.5 cm from ostium
		2) Variable narrowing 70-90% throughout vessel length
Right	1) 4 cm in length, starting adjacent to ostium. Up to 1.4 cm in diameter.	1) Complete occlusion, 2.5 cm from ostium
		2) 90% occlusion, 4.0 cm from ostium
		3) 90% occlusion, distally, at the origin of the posterior coronary artery

**Figure 2:**
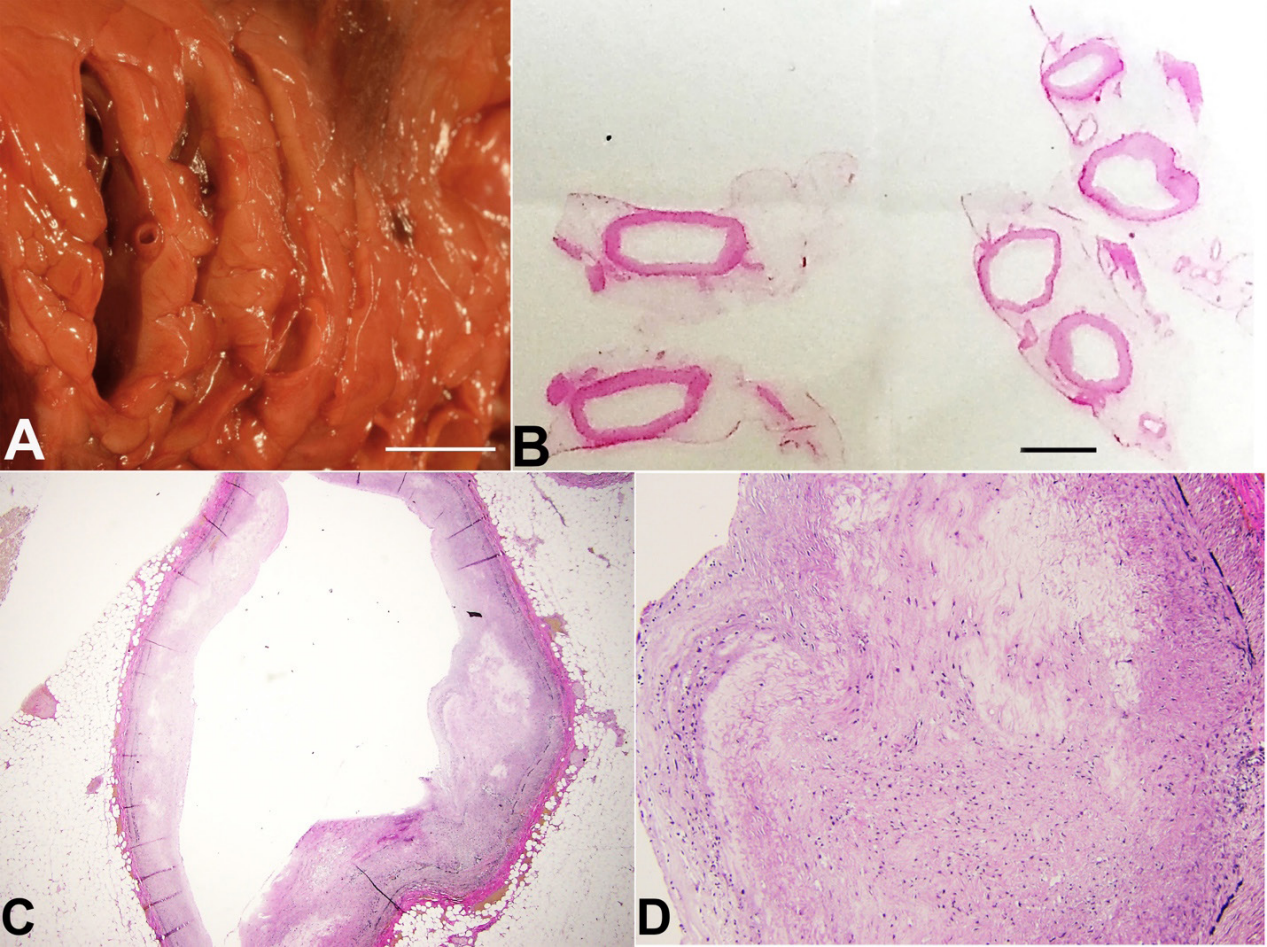
**A** – Fresh magnified gross view of the serial sections of the left anterior descending coronary artery (base of the heart and mid proximal left anterior descending coronary artery in the left side of the photo and apex and mid distal left anterior descending coronary artery in the right side of the photo) with variable degrees of coronary artery aneurysms up to 1.8 cm in diameter (scale bar= 1cm); **B** – 1 x view of microscopic slides showing dilation of the circumference of the left anterior descending coronary artery (scale bar= 1cm); **C** and **D** – 10 x and 40 x view of Verhoeff-van Gieson (VVG) stain of the left anterior coronary artery showing destruction of the tunica media with loss of smooth muscle, disarray “pale” degenerated areas, and elastic media fragmentation.

The Figure 2 shows the gross images of the left circulation with marked aneurysmal dilation and severe coronary artery atherosclerosis. The corresponding histology showed patchy destruction of the tunica media with luminal irregularities and changes in the wall caliber.

**Figure 3:**
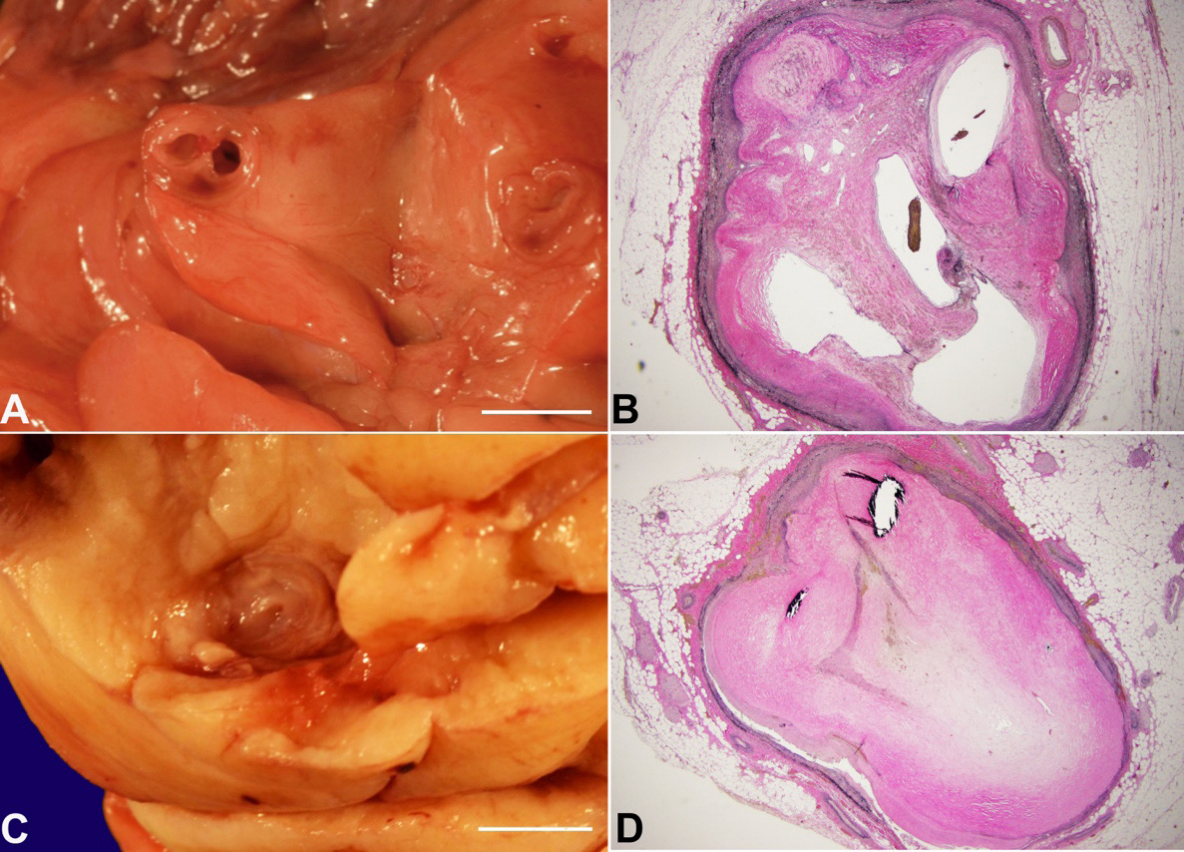
**A** – Fresh gross view of right coronary artery with aneurysmal dilation, partial luminal occlusion and recanalization (scale bar= 1 cm); **B** – 10 x view of VVG special stain of segment of the right coronary artery showing numerous small lumens lined by new endothelium, consistent with areas of old thrombosis and recanalization; **C** – Fixed gross view with complete occlusion of the left circumflex coronary artery by atherosclerotic plaque (scale bar= 1 cm); **D** – 10 x view of hematoxylin and Eosin (H&E) stained section showing left circumflex coronary artery with complete occlusion by calcified atherosclerotic plaque.

**Figure 4:**
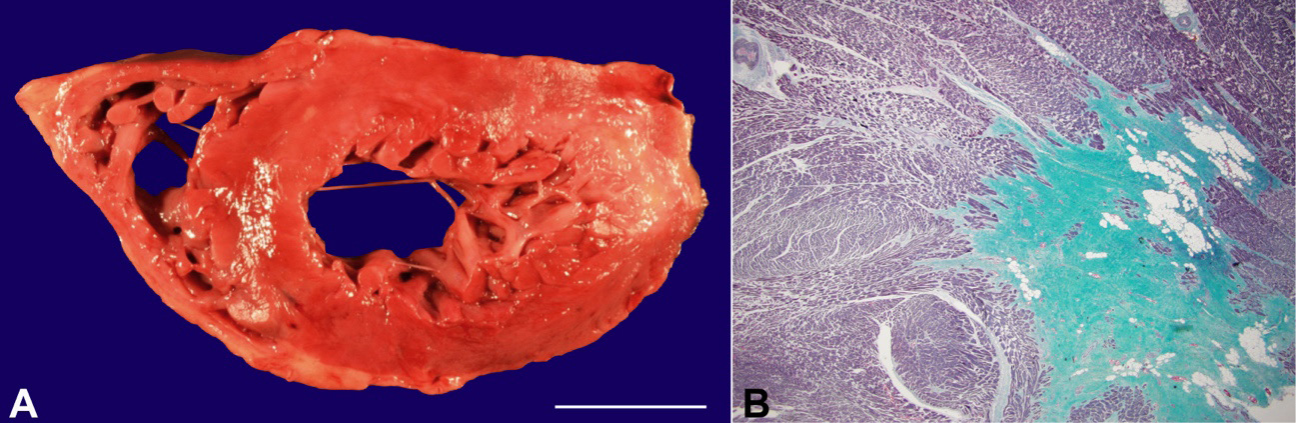
**A** – Fresh gross view of the transverse section of myocardium showing large transmural ventricular scars in the lateral/posterior left ventricle and focally in the subendocardial region of the anterior ventricle (scale bar= 3 cm); **B** – 20 x Gomori trichrome stain of myocardium (lateral/posterior aspect of the left ventricle) show extensive fibrosis consistent with remote myocardial infarcts.

The Figure 3 shows the gross images of the right circulation with histologic changes consistent with old thrombosis and recanalization. The Figure 4 shows gross images of extensive remote myocardial infarcts and histology from the lateral/posterior left ventricular wall with replacement of muscle fibers by dense collagen.

There was mild aortic atherosclerosis (focal fatty streaks) but no additional aneurysmal dilation of vessels. No dissections were present. Other autopsy findings included bilateral heavy lungs, left 600 grams (RR: 112-675 grams)^6^ and right 950 grams (RR: 155-720 grams)^6^ with an alveolar filling pattern of injury with edema and extensive neutrophil-predominant inflammatory infiltrates. The brain was involved by diffuse acute-subacute hypoxic-ischemic injury. A hematoma in the internal aspect of the sternum was present and was attributed to resuscitation efforts.

## DISCUSSION

This adult male that presented with sudden cardiac arrest while riding bicycle died due to the sequelae of remote Kawasaki disease. The mechanism of death was due to pneumonia, metabolic acidosis, and anoxic brain injury due to cardiopulmonary arrest in the setting of cardiovascular disease and coronary vascular injury, in the form of aneurysmal dilation and severe atherosclerotic disease. The manner of death was natural.

The febrile rash at age 2 that was per family reports, diagnosed as Kawasaki disease, was most likely the originating event. Unfortunately, given that the hospitalization happened three decades ago, no medical records were available for review. These limits the available information about the certainty of the original diagnosis, presenting signs and symptoms, given treatment, and ancillary testing performed.

Acute KD clinical signs include polymorphic rash, nonpurulent conjunctival injection, oropharyngeal and lip mucositis, tongue papillitis, erythema and edema of the hands and feet, as well as unilateral cervical lymphadenopathy.^1^ The most feared complication are coronary artery aneurysms (CAA), which may later lead to myocardial infarction, sudden death, or ischemic heart disease.^7^ CAA are defined as dilatations of the coronary artery exceeding 50% of the reference vessel diameter.^8^ Different etiologies have been postulated for CAA; atherosclerosis is accountable for the majority in adults, and KD in children.^9^ Other causes can be attributable to (i) inflammatory disorders such as Takayasu arteritis, giant cell arteritis, and Behcet disease; (ii) Infectious disorders like mycotic aneurysms in syphilis; (iii) connective tissue disorders such as Marfan syndrome and Ehlers-Danlos syndrome; finally, (iv) aneurysms are a known complication of angiographic procedures due to iatrogenic injury, particularly during cardiac stent placement.^10^

Microscopically, in KD, the CAA have their origin in arteritis, with neutrophils that are present seven to nine days after onset, to later be replaced by large mononuclear cells accompanied by lymphocytes and plasma cells, and fibroblastic proliferation that produces features of granulomatous arteritis. As the inflammation subsides, the tissue then becomes progressively more fibrotic, and gradually a scar is formed. The formation of CAA begins about the tenth day after onset and is completed by the twelfth day. Grossly, CAA arises from symmetrical dilatation, which itself results from dissociation and destruction of the internal elastic lamina.^11^ Lesions may persist to a chronic state, with vessel recanalization after thrombotic occlusion of the aneurysm, remodeling of vascular structures, and sometimes reocclusion, continuing to a remote stage.^12^ CAA generally remain asymptomatic, but they may become occlusive, with higher risk of myocardial infarction and sudden death. Concomitant atherosclerotic risk factors and size of the aneurysm (with giant aneurysms, defined as >0.8 cm in diameter) may determine morbidity. The aneurysms may clinically and angiographically “heal,” but it is unclear if this process involves intimal proliferation, thrombus organization, or scar formation.^13^

Intravenous immunoglobulin (IVIG) has been established as the standard-of-care treatment for KD for the last four decades due to the significant reduction in the rate of CAA as well as a reduction in the duration of fever and other symptoms associated with its use.^14^ The use of aspirin in patients with KD to reduce inflammation and prevent thrombosis through its antiplatelet effect is considered the standard of care. However, the optimal dosage is unclear.^1^

Missed KD presenting in adulthood can be suspected when CAA characteristics include proximal aneurysms with or without calcification followed by an angiographically normal distal segment. In young adults can present with ventricular tachycardia in the setting of left heart failure decades after missed KD.^15^

The only other notable risk factor present on this thin, young male was the history of hypercholesterolemia. Performed testing during the first 24 hours after admission revealed a mildly increased cholesterol, a mildly elevated LDL, mildly decreased HDL. and normal triglycerides. There is no data available for the lipid profile before admission. It has been published that the lipid profile changes during the course of hospitalization after a coronary event, but if performed within the first 3 days of admission may be reflective of the baseline of the patient.^16^ Per the family, the patient did not take any medication, but it is unclear if this is the product of non-compliance or the result of diet-only modification strategies. We don't know for how long the decedent was diagnosed with hypercholesterolemia.

It has been hypothesized that Kawasaki disease may predispose to an abnormal lipid profile with predisposition to premature atherosclerosis later in life.^17^ The relation between Kawasaki disease and hypercholesterolemia has been explored in mice models that show a pathophysiological link between coronary vasculitis and subsequent atherosclerotic acceleration.^18^ Continued long-term surveillance of children that suffered from KD may be warranted given the subsequent abnormalities in lipid metabolism.^19^ This lipid abnormalities have been found in KD patients with and without (uncomplicated KD) involvement of the coronary arteries. There are still many unknown aspects regarding the long-term prognosis of patients that suffered KD and there is still no consensus on the relationship between Kawasaki disease and atherosclerosis.^20^ Therefore, it is not entirely clear if the hypercholesterolemia was a sequalae of KD or if hypercholesterolemia acted synergically with KD as a coronary risk factor; nevertheless, current medical literature suggests an association between these two entities.

## CONCLUSION

We presented the case of an apparently healthy young adult with a sudden cardiac arrest while riding a bicycle, who had a history of febrile rash as a toddler and hypercholesterolemia. On autopsy, CAA with microscopic evidence of coronary artery injury and coronary artery atherosclerosis were present, and KD was deemed the etiology of his heart disease and his demise. The only other contributing factor to the development of atherosclerosis was hypercholesterolemia, which has been previously linked with KD. The sequence of events with chronic coronary artery injury, recanalization, thrombosis, and ischemic heart disease probably lead to arrhythmia and death. This case is of particular interest because it makes awareness about the potentially fatal consequences of KD in adulthood.
